# Assessment of an App-Based Sleep Program to Improve Sleep Outcomes in a Clinical Insomnia Population: Randomized Controlled Trial

**DOI:** 10.2196/68665

**Published:** 2025-04-23

**Authors:** Walter Staiano, Christine Callahan, Michelle Davis, Leah Tanner, Chelsea Coe, Sarah Kunkle, Ulrich Kirk

**Affiliations:** 1 Department of Physical Education and Sport Universitat de València Valencia Spain; 2 Department of Psychology University of Southern Denmark Odense Denmark; 3 Headspace Santa Monica, CA United States; 4 Fralin Biomedical Research Institute Roanoke, VA United States

**Keywords:** cognitive behavioral therapy for insomnia, mindfulness, randomized controlled trial, RCT, therapy, insomnia, behavioral, app based, app

## Abstract

**Background:**

Insomnia is the most commonly reported sleep disturbance and significantly impacts mental health and quality of life. Traditional treatments for insomnia include pharmacological interventions or cognitive behavioral therapy for insomnia (CBT-I), but these options may not be accessible to everyone who needs treatment.

**Objective:**

This study aims to assess the effectiveness of the app-based Headspace Sleep Program in adults with clinical insomnia on sleep disturbance and mental health outcomes, compared with a waitlist control group.

**Methods:**

This randomized controlled trial included 132 adults with clinical insomnia who were assigned to either the Headspace Sleep Program (an 18-session self-guided, in-app program utilizing CBT-I techniques augmented by mindfulness) or a waitlist control group. Sleep disturbance outcomes were assessed by changes in insomnia symptoms (measured using the Insomnia Severity Index) and sleep efficiency (measured via sleep diary and actigraphy). Mental health outcomes included perceived stress (measured by the 10-item Perceived Stress Scale), depressive symptoms (measured by the 8-item Patient Health Questionnaire), sleep quality (measured by the Pittsburgh Sleep Quality Index), anxiety symptoms (measured by the 7-item Generalized Anxiety Disorder Scale), and mindfulness (measured by the Mindful Attention Awareness Scale). Changes from baseline to postintervention and follow-up were assessed for each outcome.

**Results:**

Participants had a mean (SD) age of 37.2 (10.6) years, with 69 out of 132 (52.3%) identifying as female. Those randomized to the Headspace Sleep Program group experienced significantly greater improvements in insomnia symptoms from baseline to postintervention and follow-up compared with participants in the waitlist control group (P<.001, η²p=0.107). Improvements from baseline to postintervention and follow-up were also observed in the Headspace Sleep Program group for sleep efficiency, as measured by both sleep diary (P=.01, η²p=.03) and actigraphy outcomes (P=.01, η²p=.03). Participants in the Headspace Sleep Program group showed greater improvements in insomnia remission rates (8/66, 12%, at postintervention and 9/66, 14%, at follow-up) and treatment response (11/66, 17%, at postintervention and 15/66, 23%, at follow-up) compared with the control group (remission rate 2/66, 3%, at postintervention and 0/66, 0%, at follow-up; treatment response 3/66, 5%, at postintervention and 1/66, 2%, at follow-up). The results suggest significant improvements in depressive symptoms (P=.01, η²p=.04), anxiety symptoms (P=.02, η²p=.02), and mindfulness (P=.01, η²p=.03) in the Headspace Sleep Program group.

**Conclusions:**

The Headspace Sleep Program is an effective intervention for improving sleep disturbances in adults with clinical insomnia.

**Trial Registration:**

ClinicalTrials.gov NCT05872672; https://clinicaltrials.gov/ct2/show/NCT05872672

## Introduction

Sleep plays a vital role in human functioning and is essential for quality of life [1]. Sleep disorders affect the sleep quality of millions and may impact physical and mental health, increasing the risk of obesity, hypertension, heart attack, and stroke [2-8], as well as elevating stress, depression, and anxiety symptoms [9]. Insomnia, defined as difficulty initiating or maintaining sleep, is the most common sleep disorder, with 20% of adults reporting occasional insomnia symptoms and 10%-30% experiencing chronic insomnia [10,11]. In addition to its physical and mental health impacts, insomnia has significant societal and economic consequences, including reduced productivity, absenteeism, higher accident rates, increased health care costs, and an overall decline in quality of life [12]. Research suggests that insomnia imposes an annual economic burden of US $95.5 to US $107.5 billion [13].

Sleep disturbances, including insomnia, can be treated with pharmacological interventions; however, these treatments may have adverse side effects [14]. Consequently, behavioral and psychological interventions, such as cognitive behavioral therapy for insomnia (CBT-I) [15] and mindfulness [16,17], have gained popularity for treating sleep disturbances and insomnia, with a robust body of literature supporting their efficacy [15-18]. Specifically, CBT-I is the first-line treatment recommended by the American College of Physicians [19] and the European Insomnia Guidelines [20] for improving insomnia symptoms, with interventions demonstrating moderate to large and durable effects on sleep quality and sleep efficiency [21,22]. CBT-I, like traditional therapy, is most often delivered in person by a licensed mental health professional, a mode of treatment that may not be accessible to everyone in need. To improve accessibility, recent studies have explored delivering CBT-I interventions digitally, with results suggesting similar outcomes to in-person treatment [23-25]. Similarly, app-based mindfulness interventions, also offered digitally, have been shown to improve sleep quality and insomnia symptoms [16,26].

To address insomnia treatment gaps, particularly by improving accessibility (Multimedia Appendix 1), Headspace developed the Headspace Sleep Program—an 18-session, app-based, self-guided program that integrates CBT-I techniques with mindfulness. This program aims to provide individuals with insomnia an accessible way to change their relationship with sleep and adopt strategies to build and maintain sleep hygiene. While Headspace and other digital mental health companies [27-29] have developed sleep-specific content, to our knowledge, this is the first self-guided, app-based program to combine CBT-I techniques with mindfulness as a core component in a structured, 18-session sequential format. Previous research has explored mindfulness as an adjunct or alternative to CBT-I [30], with initial findings suggesting that combining the 2 interventions may enhance overall sleep outcomes [31]. However, this research still followed the traditional CBT-I structure (ie, 6 weekly sessions lasting 90-120 minutes) and was not tested in a randomized controlled trial (RCT) [31]. While mindfulness is often incorporated as part of the cognitive component of CBT-I [32], the Headspace Sleep Program positions mindfulness as a foundational approach, introducing it first as a tool for building awareness of sleep habits and patterns. This approach differs from traditional CBT-I frameworks, where mindfulness is typically introduced as a complementary cognitive strategy. Additionally, the Headspace Sleep Program integrates mindfulness-based perspectives and practices throughout the program to encourage ongoing reflection, self-compassion, and nonjudgmental awareness, further distinguishing its emphasis on mindfulness as a core rather than a supplementary component. By incorporating mindfulness as a core component, the Headspace Sleep Program aims to equip individuals with tools to address not only sleep issues but also broader concerns such as stress, depression, and anxiety symptoms, which are assessed in this study.

By integrating both CBT-I and mindfulness, the Headspace Sleep Program was designed to be more effective than mindfulness alone and more accessible than traditional CBT-I for individuals with clinical insomnia. Several features were incorporated to enhance accessibility: (1) the program is delivered via the Headspace app, offering a subscription at a lower cost than traditional CBT-I; (2) all sessions are self-guided, allowing users to access content at their convenience without relying on provider availability; (3) the intervention consists of 18 daily sessions, a significantly shorter time frame than traditional CBT-I, which is typically delivered weekly over several weeks; and (4) offering the program through the Headspace app, which may help reduce treatment stigma and increase awareness among individuals who may not realize that insomnia treatment is available to them (Multimedia Appendix 1).

This study aimed to evaluate the effectiveness of the Headspace Sleep Program in adults with clinical insomnia by assessing its impact on sleep disturbance and mental health outcomes, including perceived stress, depression, anxiety, and mindfulness. We hypothesized that participants in the Headspace Sleep Program group would show significant improvements in insomnia and mental health outcomes compared with the waitlist control group. Additionally, in an exploratory analysis, we sought to determine whether participants with mild baseline insomnia symptoms responded differently to the intervention compared with those with moderate baseline insomnia symptoms.

## Methods

### Study Design

This RCT, using a pre-post and 3-week follow-up design, evaluated the effectiveness of the Headspace Sleep Program on insomnia outcomes in 132 adults with clinical insomnia. Sample size determination was based on 2 parameters. First, the minimally important difference in insomnia severity (ie, treatment response), defined as an 8-point reduction on the Insomnia Severity Index (ISI) [10,16], with the sample size calculated using an SD of 6 points based on previous studies [33,34]. Second, a priori power calculations using G*Power (Heinrich-Heine-Universität Düsseldorf) indicate that, with a sample size of 108, our study had 80% power to detect significant (*P*<.05) between-within interaction effects (effect size *f*=0.12), corresponding to a small-to-medium effect size by ANOVA (up to 3-way) [35]. In both parameters, we accounted for a 20% attrition rate. Participants completed a 7-day baseline period in which they maintained their normal sleep patterns while wearing an actigraphy watch during sleep and completing a sleep diary the following morning. After the baseline period, participants completed the baseline sleep disturbance and mental health questionnaires. The randomization procedure was determined after study recruitment but before the study launch. Specifically, participants were randomly allocated to 1 of 2 groups: the Sleep Program or the waitlist control. The research team was not formally blinded to group allocation. Participants were not informed of their group assignment until after completing the baseline assessment. Those in the Headspace Sleep Program were instructed to follow the 18-session program daily, while those in the waitlist control group were asked to maintain their regular routines and were granted access to the Sleep Program after the study.

After the intervention, participants were reassessed using the sleep disturbance and mental health questionnaires before completing a 7-day postintervention period. During this period, as in the baseline phase, they maintained their normal sleep patterns while wearing an actigraphy watch during sleep and completing a sleep diary the following morning. Participants were instructed not to access the app-based Sleep Program from the time of the postintervention assessment for the remainder of the study. Deidentified in-app user data for each participant were extracted by the study team to assess adherence to this requirement. Three weeks later, participants completed a final 7-day follow-up period with actigraphy, a sleep diary, and the questionnaires.

### Ethical Considerations

Study procedures were approved by the Institutional Review Board at Virginia Tech (approval number 21-295). Participants signed a consent form before enrolling in the study and were informed that they could withdraw at any time. All data were deidentified before analysis.

### Participants

Participants were recruited through flyers and advertisements at a local university (Virginia Tech, Virginia, USA). Inclusion criteria were assessed during the screening phase and included meeting the insomnia research diagnostic criteria (ISI score ≥11) [36], being 18-65 years old, and having access to a smartphone. Participants using sleep medications were included if their self-reported dosage had been stable for at least 6 weeks (medication dosage and type were balanced between study groups). Exclusion criteria, also self-reported and assessed during the screening phase, included (1) risk factors that may be comorbid with severe insomnia (eg, depression and self-harm suspected to interfere with the study protocol or requiring immediate treatment), assessed using the question, “The thought of harming myself has occurred to me,” and the 8-item Patient Health Questionnaire (PHQ-8) [37]. Participants scoring >0 (range 0-3) on the self-harm screening question and >20 on the PHQ-8 screening questionnaire were not included in the study. For safety reasons, individuals meeting these criteria were referred to mental health resources (National Alliance on Mental Illness helpline). Additional exclusion criteria included the following: (2) enrollment in a CBT-I program within the past 6 months; (3) regular mindfulness meditation practice (≥10 minutes, ≥2 times per week); and (4) inadequate English proficiency. Participants were compensated for their participation according to the following structure: US $50 for completing the baseline assessment, US $50 for the postintervention assessment, US $50 for the follow-up measures, and a US $100 completion bonus for completing >75% of the content. While compensation was intended to encourage participants to engage with >75% of the app content, it may have also limited the generalizability of the findings by contributing to higher adherence rates in this study.

### Headspace Sleep Program (Intervention)

The Headspace Sleep Program is an app-based, self-guided program that incorporates CBT-I techniques augmented by mindfulness to help individuals adopt a nonjudgmental, self-compassionate approach to improving sleep habits. The 18-session program, designed for daily completion, includes mindfulness principles, cognitive techniques, relaxation techniques, and CBT-I behavioral techniques (eg, sleep hygiene, stimulus control, and wind-down routines). Notably, to enhance approachability and safety, the program excludes traditional CBT-I sleep restriction protocols. Instead, it provides information on the concept of sleep restriction and suggests it as an option for those who wish to try it. Sessions 1-11 are considered the core elements of the program and are assumed to provide the minimum effective dose, as all key techniques (eg, mindfulness, CBT-I, relaxation) are introduced by this point. Sessions 12-18 focus on integrating these techniques into daily practice. The content is delivered through short videos (5-10 minutes per day) in the Headspace app, and users are encouraged to engage in small daily actions (eg, trying a new wind-down routine). To enhance engagement, the program includes animations and videos featuring mindfulness teachers, sleep experts, and individuals with sleep difficulties. A supplemental workbook helps users track sleep, reflect on daily experiences, and create personalized sleep plans. Participants received instructions for app usage and followed the program without an introductory session to best reflect real-world engagement. They had the option to set up the app during the initial laboratory visit and were encouraged to contact the study team if they encountered technical issues related to app setup or usage. None of the participants reported technical difficulties when setting up the app in the laboratory, at home, or during the study. Multimedia Appendix 2 outlines and describes all sessions in the Headspace Sleep Program.

### Waitlist Control

The waitlist control group was instructed to maintain their regular routines and was given access to the Headspace Sleep Program after study completion.

### Insomnia Outcomes

The ISI was used to assess self-reported insomnia symptoms at screening, baseline, postintervention, and the 3-week follow-up (score range 0-28; higher scores indicate more insomnia symptoms; subgroups: score range for moderate insomnia 15-21, score range for severe insomnia 22-28; Cronbach α=0.87) [38,39]. Additionally, the ISI has been validated as a clinical end point for a minimally important treatment response (ISI total score reduction >7) and remission (ISI total score <8) [38,39]. Total scores and clinical end points (ie, treatment response and remission) were assessed.

Subjective sleep quality was measured using a sleep diary, which calculated the following:

- Sleep efficiency (SE, %): the percentage of the sleep period spent sleeping (duration of sleep/duration of time in bed);
- Sleep onset latency (SOL; minutes): the duration of time from when the lights are turned off until falling asleep;
- Wake after sleep onset (WASO; minutes): the minutes awake after initially falling asleep; and
- Total sleep time (TST; minutes): the amount of sleep obtained at night [40].

The sleep diary was completed every morning over a period of 7 days at 3 time points (baseline, postintervention, and 3-week follow-up). For each time point, sleep diary data were averaged across the 7-day period, with a minimum of 4 days required for inclusion.

Objective sleep quality was measured using wrist actigraphy, a valid measure commonly used to assess sleep patterns [41]. Sleep was measured with the ActiGraph wGT3X-BT accelerometer at a frequency of 50 Hz (ActiGraph Corporation). Participants were instructed to wear the watch on their nondominant wrist from 2 hours before bedtime until the next morning for 7 consecutive days, at the same 3 time points as the sleep diary. Identical sleep parameters were computed for actigraphy as for the sleep diary (SE, SOL, WASO, and TST). For all 3 time points, data were averaged across the 7-day period, with a minimum of 4 days required for inclusion. Sleep patterns were assessed using a previously validated software algorithm based on the Cole-Kripke scoring method [42] and processed with ActiLife software (version 6.13.5; ActiGraph Corporation).

### Mental Health Outcomes

All mental health questionnaires were collected at baseline, postintervention, and the 3-week follow-up. Perceived stress was assessed using the 10-item Perceived Stress Scale (PSS-10) (total score range 0-40, with higher scores indicating higher perceived stress; Cronbach α=0.85) [43,44]. The 8-item PHQ was used to measure depressive symptoms (total score range 0-24; higher scores indicate more depressive symptoms; Cronbach α=0.86) [37]. The Pittsburgh Sleep Quality Index (PSQI) was used to assess sleep quality (total score range 0-21, where “0” indicates no sleep difficulty and “21” indicates severe sleep difficulties [45]; Cronbach α=0.83) [45]. The 7-item Generalized Anxiety Disorder (GAD-7) was completed to measure anxiety symptoms (total score range 0-21, with higher scores indicating more anxiety symptoms; Cronbach α=0.89) [46]. Mindfulness was assessed using the Mindful Attention Awareness Scale (MAAS) (mean of the 15 items, range 1-6, with higher scores indicating higher levels of trait mindfulness; Cronbach α=0.80) [47]. The MAAS was chosen because it has been used in previous studies assessing the effects of mindfulness on sleep quality [48,49]. At postintervention, potential adverse effects were assessed by asking participants to report the number of potential adverse effects experienced during the intervention period. Specifically, during the postintervention in-person visit, we asked participants, “Did you experience any adverse effects as a participant in the app-based intervention?” Participants were given the option to write down any comments or responses to this question. No adverse effects were reported. Usage of sleep medication in both the intervention group and the waitlist control group was reported at baseline and follow-up assessments.

Adherence to the Headspace Sleep Program was obtained from in-app user data for each participant. It was calculated by summing the total number of completed sessions in the Headspace Sleep Program during the intervention period.

### Statistical Analysis

All analyses were conducted using the intention-to-treat (ITT) and study-completer approaches. Study-completer results, shown in Multimedia Appendix 3, are comparable to the ITT analyses. For the ITT analysis, we used multiple imputations to handle missing outcomes (15/132, 11.4%, values were missing at postintervention and follow-up). Multiple imputations (10 imputations) were used to achieve a relative efficiency of 99%, ensuring in-range values.

Assumptions of statistical tests for normal distribution and sphericity of data were checked using the Shapiro-Wilk test, histograms, Q-Q plots, and box plots. A series of mixed 2 (groups: intervention and control) × 3 (time: preintervention, postintervention, and follow-up) ANOVAs was performed on the ISI, sleep diary variables, actigraphy variables, PSS-10, PSQI, PHQ-8, GAD-7, and MAAS. To analyze insomnia subgroups, a series of mixed 2 (groups: intervention and control) × 3 (time: preintervention, postintervention, and follow-up) × 2 (subgroups: mild insomnia and severe insomnia) ANOVAs was performed on the ISI, PSS-10, PSQI, PHQ-8, GAD-7, and MAAS. To examine clinical end points, logistic regression analyses were conducted to test differences in treatment response and remission using the cut scores described above on the ISI across postintervention and follow-up. Logistic regression analyses on remission and response status were performed to compare the 2 groups (intervention and control) at postintervention and 3-week follow-up (outcome variables) in relation to preintervention values (covariate), without stepwise regression.

All significant interactions were followed up with relevant corrected pairwise comparisons using the Bonferroni method for simple main effects within each group. Where no significant interactions were found, the main effects of time and group were reported. Significance was set at .05 (2-tailed) for all analyses, and ANOVA effect sizes were calculated as partial eta squared (η²p), with 0.02, 0.13, and 0.26 indicating small, medium, and large effects, respectively. Pearson correlations were used to assess whether a dosage-response relationship was present. Data analysis was conducted using SPSS (version 27; IBM Corp).

## Results

### Participant Enrollment and Intervention Adherence

**Figure 1:**
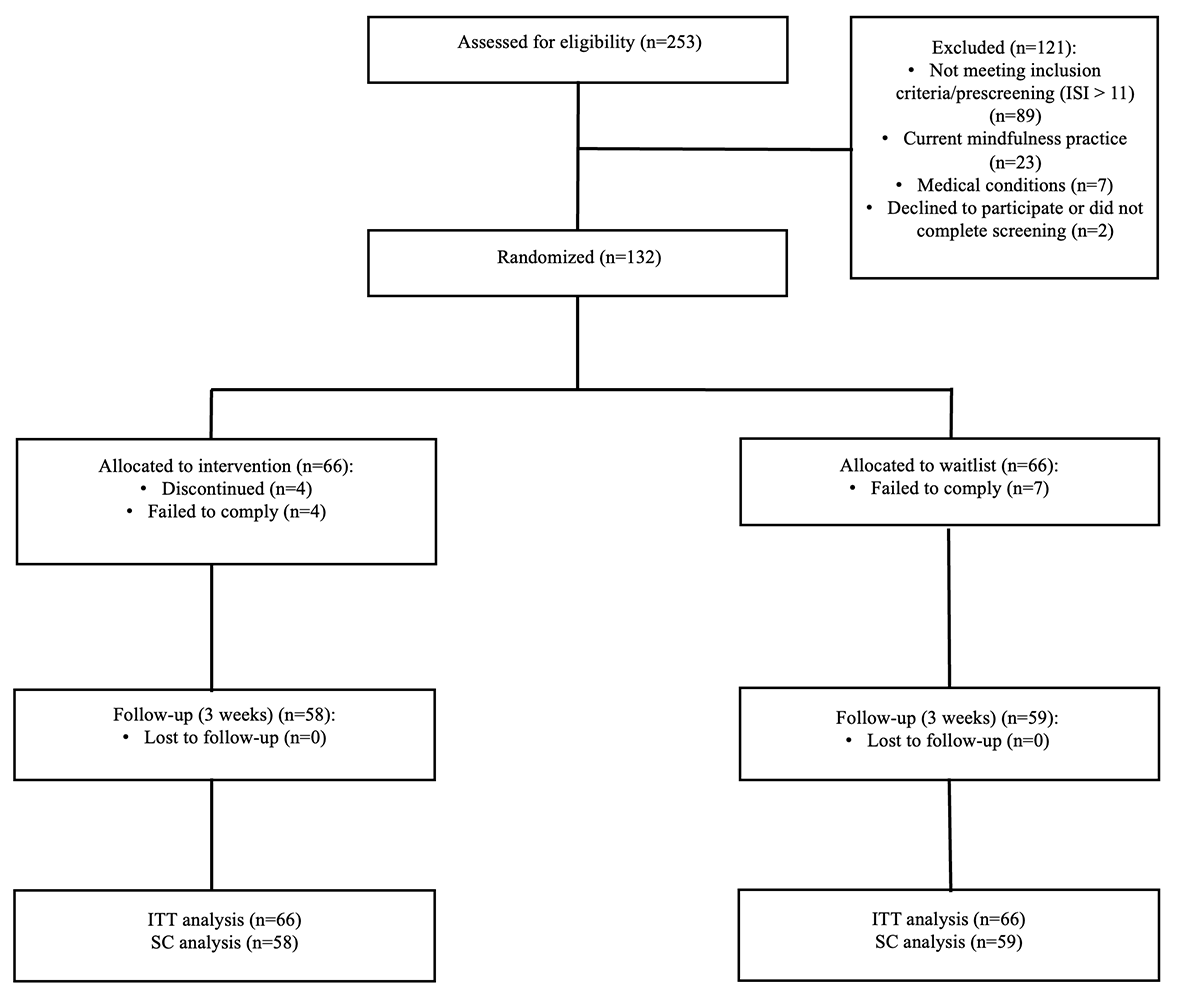
CONSORT (Consolidated Standards of Reporting Trials) diagram and recruitment flowchart. ISI: Insomnia Severity Index; ITT: intention-to-treat analysis; SC: study completer analysis.

Overall, 253 potential participants were assessed for eligibility, with 132 meeting the inclusion criteria, completing the informed consent process, and enrolling in the study (Figure 1; also see Multimedia Appendix 4). The 132 participants included in the analysis had a mean (SD) age of 37.2 (10.6) years, and 69 (52.3%) females identified as female (Table 1). No significant differences (all *P*s>.32; discussed later) were found between the intervention and control groups at baseline for demographic characteristics or study outcomes. On average, participants in the intervention group completed 13.0 (SD 5.4) sessions, corresponding to 13 of the 18 (72%) intervention contents. Of the 66 participants, 65 (98%) completed at least two sessions of the app-based Sleep Program. Additionally, 24 (36%) participants completed all 18 sessions of the intervention and 51 (77%) completed at least half of the intervention.

**Table 1:** Demographics and clinical characteristics by study group of the intention-to-treat sample.

Characteristics		Sleep intervention (n=66)	Waitlist control (n=66)
**Gender, n (%)**			
	Male	32 (48)	31 (47)
	Female	34 (52)	35 (53)
	Age, mean (SD)	37.3 (11.5)	37.0 (9.6)
**Marital status, n (%)**			
	Married	26 (39)	24 (36)
	In a relationship	14 (21)	14 (21)
	Single	18 (27)	22 (33)
	Divorced	8 (12)	6 (9)
**Employment, n (%)**			
	Full time	35 (53)	32 (48)
	Part time	17 (26)	14 (21)
	Retired	2 (3)	4 (6)
	Unemployed	12 (18)	16 (24)
**Household income (US $), n (%)**			
	<20,000	9 (14)	9 (14)
	20,000-35,000	12 (18)	14 (21)
	35,001-50,000	14 (21)	13 (20)
	50,001-75,000	11 (17)	11 (17)
	75,001-100,000	9 (14)	10 (15)
	>100,000	11 (17)	9 (14)
**Ethnicity, n (%)**			
	White	34 (52)	32 (48)
	Asian	7 (11)	11 (17)
	Black	10 (15)	12 (18)
	Hispanic	9 (14)	7 (11)
	Other	6 (9)	4 (6)
**Sleep medication, n (%)**			
	Yes (baseline)	17 (26)	17 (26)
	No (baseline)	49 (74)	49 (74)
	Yes (follow-up)	18 (27)	18 (27)
	No (follow-up)	48 (73)	48 (73)

At baseline, medication status in the intervention group showed that 17 of the 66 (26%) participants used sleep medication regularly, while 49 of the 66 (74%) participants did not. In the control group, 18 of the 66 (27%) participants used sleep medication regularly at baseline, whereas 48 of the 66 (73%) did not. At follow-up, there was no change in sleep medication usage in either group. There were no differences between the 2 groups at baseline or follow-up, and no changes within either group (Table 1). Medication status was used as a control variable to assess its impact on the outcomes. No significant interactions or main effects of medication status were observed for any variable analyzed (*P*=.61).

### Insomnia Outcomes

ISI results indicated a group × time interaction (*P*<.001, η²p=0.107), with the Headspace Sleep Program group showing a significant decrease in the ISI scores from pre- to postintervention (from a mean score of 14.8 to a mean score of 11.95, 19.26% decrease; *P*<.001), from preintervention to follow-up (from a mean score of 14.8 to a mean score of 10.70, 27.70% decrease; *P*<.001), and from postintervention to follow-up (from a mean score of 11.95 to a mean score of 10.70, 10.46% decrease; *P*=.02). No significant changes were observed in the control group (*P*=.35; Table 2).

**Table 2:** Behavioral data by study group and time point of the intention-to-treat sample.

Clinical value	Sleep intervention (n=66)				Waitlist control (n=66)		
	Pre, mean (SD)	Post, mean (SD)	Follow-up, mean (SD)	Pre, mean (SD)		Post, mean (SD)	Follow-up, mean (SD)
Insomnia Severity Index	14.80 (3.13)	11.95 (3.93)^a,b^	10.70 (3.37)^b,c,d,e^	14.81 (2.93)		14.88 (4.60)	14.80 (3.39)
Pittsburgh Sleep Quality Index	10.31 (2.48)	9.41 (2.37)^a,b^	8.12 (2.43)^b,c,d,e^	10.43 (2.58)		10.64 (2.25)	10.18 (1.83)
10-item Perceived Stress Scale	16.89 (3.91)	18.17 (3.59)	17.73 (3.63)	17.09 (3.65)		17.84 (3.06)	17.97 (2.49)
8-item Patient Health Questionnaire	11.42 (3.69)	10.03 (4.52)^a,b^	9.01 (3.87)^b,c,d,e^	11.56 (3.37)		11.41 (2.78)	10.72 (2.97)
7-item Generalized Anxiety Disorder	10.89 (4.40)	8.76 (3.84)^a,b^	8.21 (4.25)^b,c,d,f^	10.80 (3.89)		11.27 (3.57)	11.08 (3.52)
Mindful Attention Awareness Scale	50.28 (15.38)	52.83 (15.11)^a,e^	54.80 (14.40)^b,c,d,e^	50.69 (13.89)		50.35 (12.28)	50.83 (11.28)

^a^Significant difference between pre and post time points.

^b^*P*<.001.

^c^Significant difference between pre and follow-up time points.

^d^Significant difference between post and follow-up time points.

^e^*P*<.01.

^f^*P*<.05.

Remission rates (ISI total score <8) [38,39] in the intervention group increased significantly (*P*=.03) over time (8/66, 12%, at postintervention and 9/66, 14%, at follow-up). By contrast, remission rates in the control group remained unchanged (*P*=.29; 2/66, 3%, at postintervention and 0/66, 0%, at follow-up; Multimedia Appendix 5). Similarly, treatment response (ISI total score reduction >7) [38,39] showed a steady increase from pre- to postintervention (11/66, 17%) to follow-up (15/66, 23%) in the intervention group. By contrast, treatment response did not significantly change in the control group (*P*=.27; 3/66, 5%, at postintervention and 1/66, 2%, at follow-up; Multimedia Appendix 6).

Sleep diary results suggest that the Headspace Sleep Program group significantly increased SE from pre- to postintervention (*P*=.002) and from preintervention to follow-up (*P*=.01), but not from postintervention to follow-up (*P*=.69; Table 3). A significant group × time interaction was identified (*P*=.01, η²p=0.05). No significant changes in SE were observed in the control group (*P*=.19). No group × time interactions were found for SOL (*P*=.15, η²p=.01), WASO (*P*=.18, η²p=.01), and TST (*P*=.11, η²p=.01). For TST, a significant main effect of group (*P*=.04, η²p=.03) indicated that the groups were significantly different, regardless of time points (pre-, postintervention, or follow-up; Table 3). No main effects of time were observed for SOL (*P*=.15, η²p=.01) or WASO (*P*=.38, η²p=.01). A significant main effect of group for SOL (*P*=.03, η²p=.04) showed that both groups increased in SOL from pre- to postintervention and follow-up (Table 3).

**Table 3:** Subjective measures of sleep (ie, sleep diary) by study group and time point of the intention-to-treat sample.

Clinical value	Sleep intervention (n=66)				Waitlist control (n=66)		
	Pre, mean (SD)	Post, mean (SD)	Follow-up, mean (SD)	Pre, mean (SD)		Post, mean (SD)	Follow-up, mean (SD)
Sleep efficiency (%)	75.21 (16.51)	82.38 (9.98)^a,b^	82.01 (13.10)^c,d^	77.10 (12.84)		75.04 (14.24)	78.29 (9.58)
Sleep onset latency (minutes)	39.66 (21.97)	29.45 (23.31)	26.97 (29.71)	38.33 (28.23)		42.08 (39.76)	35.92 (25.14)
Wake after sleep onset (minutes)	85.59 (83.53)	60.40 (53.97)	69.43 (77.78)	75.74 (69.17)		82.22 (65.93)	70.90 (62.75)
Total sleep time (minutes)	383.93 (104.59)	402.65 (78.64)	410.53 (104.67)	390.98 (101.91)		378.67 (95.34)	369.41 (86.64)

^a^Significant difference between pre and post time points.

^b^*P*<.05.

^c^*P*<.01.

^d^Significant difference between pre and follow-up time points.

Objective sleep quality actigraphy results mirrored those of the subjective sleep diary. A group × time interaction was identified for SE (*P*<.001, η²p=.085), SOL (*P*<.001, η²p=.06), and WASO (*P*=.01, η²p=.03), but not for TST (*P*=.06, η²p=.02). The Headspace Sleep Program group showed significant improvements in SE, SOL, and WASO (*P*=.01), with no significant improvements observed in the control group (*P*=.25; Table 4). For TST, a significant main effect of group (*P*=.02, η²p=.04) indicated a significant difference between the groups, regardless of time points (Table 4).

**Table 4:** Objective measures of sleep (ie, actigraphy) by study group and time point of the intention-to-treat sample.

Clinical value	Sleep intervention (n=66)				Waitlist control (n=66)		
	Pre, mean (SD)	Post, mean (SD)	Follow-up, mean (SD)	Pre, mean (SD)		Post, mean (SD)	Follow-up, mean (SD)
Sleep efficiency (%)	74.40 (7.17)	82.20 (8.26)^a,b^	81.85 (9.93)^b,c^	76.49 (9.61)		74.61 (13.04)	75.08 (11.12)
Sleep onset latency (minutes)	50.34 (22.23)	31.36 (20.58)^a,b^	28.15 (21.98)^b,c^	39.89 (23.39)		43.57 (38.30)	42.83 (24.98)
Wake after sleep onset (minutes)	78.71 (45.94)	56.92 (45.08)^a,d^	62.15 (53.62)^c,d^	67.60 (48.58)		82.65 (64.63)	73.14 (46.95)
Total sleep time (minutes)	377.71 (58.49)	395.52 (52.14)	391.80 (60.90)	383.30 (69.51)		374.54 (89.25)	354.60 (70.32)

^a^Significant difference between pre and post time points.

^b^*P*<.001.

^c^Significant difference between pre and follow-up time points.

^d^*P*<.05.

Subgroup analysis compared mild versus moderate baseline insomnia symptoms (mild: ISI range 8-14, 63 patients; moderate: ISI range 15-21, 69 patients). ANOVA results showed a significant 3-way interaction (*P*=.02, η²p=.03). Follow-up testing revealed that participants with moderate baseline insomnia symptoms in the Headspace Sleep Program group experienced significant improvements in the ISI from pre- to postintervention (*P*<.001), preintervention to follow-up (*P*<.001), and postintervention to follow-up (*P*=.03; Multimedia Appendix 7). Participants with mild baseline insomnia symptoms in the Headspace Sleep Program group did not show a significant change from preintervention to postintervention (*P*=.79). However, they did show significant improvements from preintervention to follow-up (*P*=.04) and from postintervention to follow-up (*P*=.04).

### Mental Health Outcomes

Group × time interactions were identified for the PHQ-8 (*P*=.01, η²p=.03), PSQI (*P*<.001, η²p=.10), GAD-7 (*P*<.001, η²p=.11), and MAAS (*P*=.01, η²p=.04). Results for the PHQ-8, PSQI, GAD-7, and MAAS showed that the Headspace Sleep Program group significantly improved at all time points (*P*=.02), whereas the control group did not show significant changes at any time point (*P*=.19). PSS-10 results did not show any group × time interaction (*P*=.63, η²p=.005) or main effect of group (*P*=.42, η²p=.001). However, a significant main effect of time (*P*=.01, η²p=.03) indicated that both groups showed an increase in PSS-10 scores over time, from pre- to postintervention to follow-up (Table 2).

Subgroup ANOVA results showed no significant 3- or 2-way interactions for the PSS-10 (*P*=.41, η²p=.003). However, a main effect of time was observed (*P*=.005, η²p=.03). Similarly, no significant 3- or 2-way interactions were found for the PHQ-8 (*P*=.33, η²p=.004). A main effect of time (*P*<.001, η²p=.05) and group (*P*=.007, η²p=.04) was observed. No significant 3- or 2-way interactions were found for the PSQI (*P*=.43, η²p=.002). A main effect of time was observed (*P*=.02, η²p=.03). No significant 3- or 2-way interactions were found for GAD-7 (*P*=.27, η²p=.001). A main effect of time (*P*<.001, η²p=.05) and group (*P*=.01, η²p=.04) was observed.

ANOVA results showed a significant 3-way interaction for the MAAS (*P*=.02, η²p=.03), with follow-up testing revealing that the moderate insomnia group in the experimental condition showed significant improvement in the MAAS scores from pre- to postintervention (*P*=.003) and from preintervention to follow-up (*P*<.001), but not from postintervention to follow-up (*P*=.20). By contrast, the mild insomnia group in the experimental condition did not show a significant change from preintervention to postintervention (*P*=.60) or from preintervention to follow-up (*P*=.08). However, a significant difference was observed from postintervention to follow-up (*P*=.03). No significant differences were found for either moderate (*P*=.31) or mild insomnia (*P*=.35) in the waitlist control group at any time point. Significant dosage-response correlations (*P*=.02) were found with weak to moderate strength (ranging from 0.28 to 0.34) only for SE at postintervention (for the sleep diary variable) and WASO at postintervention (for the actigraphy variable). No other variables showed significant differences (*P*=.23).

## Discussion

### Principal Findings

This study aimed to evaluate the efficacy of the Headspace Sleep Program in improving sleep disturbances and mental health outcomes in adults with clinical insomnia. The results provide support for the study hypotheses, showing that insomnia symptoms significantly decreased and both subjective and objective sleep quality, as well as mental health outcomes, significantly improved for participants in the Headspace Sleep Program group compared with those in the waitlist control group. Importantly, the program’s benefits were maintained 3 weeks postintervention. Clinically, participants in the Headspace Sleep Program group exhibited higher remission rates and better treatment responses compared with those in the control group, although the effect sizes were small.

The findings of this study contribute to existing research supporting the efficacy of CBT-I techniques and mindfulness as nonpharmacological treatments for insomnia symptoms [16,23-26]. Importantly, this clinical trial extends the literature by demonstrating that an app-based, self-guided sleep program incorporating CBT-I techniques augmented with mindfulness is an effective intervention for reducing insomnia symptoms. The results of this trial suggest that the Headspace Sleep Program could serve as a viable alternative for improving sleep and mental health in individuals unable to access in-person or alternative CBT-I treatments. While CBT-I remains the first-line treatment for insomnia, as endorsed by the American College of Physicians [19] and the European Insomnia Guidelines [20], it may not be accessible to everyone in need of treatment. The results of this trial suggest that the Headspace Sleep Program could serve as a viable alternative for improving sleep and mental health in individuals unable to access in-person or alternative CBT-I treatments. As such, digital CBT-I interventions have been developed, suggesting outcomes similar to in-person treatment [23-25]. However, digital CBT-I can still be inaccessible to those who need it due to its cost, reliance on a provider’s schedule, and the fact that treatment can take weeks to show results [18]. The Headspace Sleep Program was designed to be lower in cost, more timely, and more flexible compared with traditional CBT-I. Results suggest that the program improves insomnia symptoms and mental health while also being more accessible than traditional treatments (Multimedia Appendix 1). Specifically, the Headspace Sleep Program is more accessible than both in-person and digital CBT-I because (1) the program is delivered via the Headspace app, which offers a subscription at a lower cost than both digital and in-person CBT-I; (2) all sessions are self-guided, allowing patients to access content at their convenience without relying on providers’ availability; (3) the intervention consists of 18 daily sessions, a much shorter time frame than traditional CBT-I, which is typically delivered weekly over several weeks; and (4) offering the program through the Headspace app may reduce treatment stigma and make it available to those who may not be aware that insomnia treatment is accessible to them (Multimedia Appendix 1). For these reasons, we note that the program was not designed to be a digital CBT-I intervention, but rather to use CBT-I techniques augmented by mindfulness, creating a program that is more effective than mindfulness-focused sleep content while being more accessible than traditional CBT-I. As such, research on digital CBT-I suggests greater remission rates compared with the current program [32,50]. Specifically, the SHUTi CBT-I intervention reported a remission rate of 56.7% (vs the current study’s 9/66, 14%) [16]. However, the SHUTi CBT-I involved 9 weeks of clinical sessions, whereas the current program consisted of 18 consecutive self-guided sessions. Our study’s findings suggest results that are similar to or stronger than those from interventions using mindfulness alone to improve insomnia [16]. Therefore, the Headspace Sleep Program’s design, which combines CBT-I techniques with mindfulness, may be more beneficial than mindfulness alone, and its greater accessibility could provide an effective intervention for individuals who are unable to receive traditional CBT-I to improve sleep quality.

This study also evaluated the efficacy of the Headspace Sleep Program on mental health outcomes, including perceived stress, depression, anxiety, and mindfulness. The results suggest that the intervention led to improvements in depressive and anxiety symptoms, as well as in mindfulness. On average, participants entered the study with mild depressive symptoms and moderate anxiety symptoms. While this study cannot fully disentangle the confounding relationship between participants’ baseline insomnia, depressive, and anxiety symptoms, or determine whether the program’s impact on insomnia symptoms directly caused the decrease in depressive and anxiety symptoms, the findings suggest that mental health outcomes significantly improved for those who engaged in the program. In addition, the PSQI showed a significant improvement in sleep quality in the intervention group, supporting the ISI, sleep diary, and actigraphy results. However, the results did not show improvements in perceived stress, despite the improvements in depressive and anxiety symptoms and the comorbidity between these disorders [51]. A possible explanation for the nonsignificant perceived stress result may be situational circumstances driving overall stress. The relationship between sleep and stress is bidirectional and complex. Our app-based intervention focused primarily on sleep, which might not have adequately addressed the multifaceted nature of stress, often involving psychosocial factors [52]. However, our app-based intervention did incorporate mindfulness, and previous studies have demonstrated significant reductions in self-reported stress using app-based mindfulness interventions in participants with elevated stress [53]. Future studies are needed to further disentangle the impact of app-based sleep interventions on stress. Trait mindfulness increased in the intervention group, which may be attributed to the mindfulness components embedded within the Headspace Sleep Program. This finding supports previous research suggesting that mindfulness is effective in patients with chronic insomnia [16,17].

As is typical with digital programs, there was a dropout from the intervention (15/132, 11.4%, values were missing at post and follow-up); however, ITT analyses still identified significant improvements. Sensitivity and missing data analyses did not alter the study conclusions when compared with study-completer analyses (Multimedia Appendix 3).

There were no changes in sleep medication usage in either the intervention group or the waitlist control group from baseline to follow-up. This nonsignificant finding may be explained by the relatively short period between baseline and follow-up (approximately 6 weeks). Regarding adherence rates in this study, participants, on average, completed 13 of the 18 (72%) intervention contents. Previous studies using digital sleep interventions have found comparable results. Specifically, one study reported that participants completed, on average, 50% of the intervention [54], another found a completion rate of 56.7% [55], and a third study reported 93.3% adherence [56]. Thus, our study demonstrates adherence rates similar to those observed in other app-based sleep intervention trials.

### Strengths, Limitations, and Future Research

To our knowledge, this is the first RCT to investigate the impact of an app-based, self-guided program that uses CBT-I techniques augmented by mindfulness to improve sleep disturbance in adults with clinical insomnia. The adherence data suggest that the intervention is feasible, while both subjective and objective sleep and mental health outcomes indicate that the intervention is effective. However, it should be noted that the financial compensation provided to participants may have inflated adherence rates. Other limitations of the study include the potential impact of response bias on the self-report survey results. Specifically, because participants were compensated, we cannot rule out the possibility that their responses to participant-reported outcomes may have been biased. However, as the study also used objective measures, it reduced the likelihood of bias. Additionally, the requirement for participants to have smartphone access may have impacted the overall sample, making the results less generalizable. Ethical guidelines, such as those from the Council for International Organizations of Medical Sciences, recommend that an established effective intervention be offered to study participants in waitlist control groups, which was not adhered to in this study. However, all participants in the waitlist control group were given the option to access the Headspace Sleep Program after study completion. Furthermore, although group randomization was performed before the study launch, the research team was not formally blinded, and thus, allocation concealment may have introduced biased estimates of intervention effects, as shown in previous research [57]. Finally, this study used a waitlist control group; future research should include comparisons to sleep-specific mindfulness content or other traditional insomnia treatments. Future research could expand on this study by evaluating the Headspace Sleep Program in a real-world setting and investigating the integration of the Headspace Sleep Program with a Headspace mental health coach. Research shows that pairing digital mental health interventions with human support may improve adherence and overall outcomes [58].

### Conclusions

This RCT found that the Headspace Sleep Program significantly improved insomnia symptoms, sleep quality, sleep disturbance, depressive symptoms, anxiety symptoms, and mindfulness. Insomnia remission rates and treatment response increased over time in the Headspace Sleep Program group, but not in the waitlist control group. These results suggest that the program is an effective intervention for improving sleep disturbance in adults with clinical insomnia. However, further research is needed to better understand the impact of the Headspace Sleep Program compared with traditional treatments and in real-world settings.

## Appendix

Multimedia Appendix 1 Differences between the Headspace Sleep Program, sleep mindfulness content, and cognitive behavioral therapy for insomnia.

Multimedia Appendix 2 Headspace Sleep Program session outline and descriptions.

Multimedia Appendix 3 Supplemental results.

Multimedia Appendix 4 CONSORT-EHEALTH (V 1.6.1) checklist.

Multimedia Appendix 5 Percentage of participants who met the criteria for treatment remission (ISI score<8 at both postintervention and follow-up). ISI: Insomnia Severity Index. Figure S1

Multimedia Appendix 6 Percentage of participants who met the criteria for a minimally important treatment response (ISI total score reduction from baseline >7 points at postintervention and follow-up). ISI: Insomnia Severity Index.

Multimedia Appendix 7 Subgroup analysis comparing baseline mild versus moderate insomnia symptoms. $ indicates 3-way interaction.

## Abbreviations

CBT-I: cognitive behavioral therapy for insomnia
GAD-7: 7-item Generalized Anxiety Disorder
ISI: Insomnia Severity Index
ITT: intention-to-treat
MAAS: Mindful Attention Awareness Scale
PHQ-8: 8-item Patient Health Questionnaire
PSQI: Pittsburgh Sleep Quality Index
PSS-10: 10-item Perceived Stress Scale
RCT: randomized controlled trial
SOL: sleep onset latency
TST: total sleep time
WASO: wake after sleep onset
