# Supplementary material for: Assessment of an App-Based Sleep Program to Improve Sleep Outcomes in a Clinical Insomnia Population: Randomized Controlled Trial
Source: JMIR Mhealth Uhealth. 2025 Apr 23;13:e68665. doi: 10.2196/68665 (PMC12059489; doi:10.2196/68665)
Supplement: Multimedia Appendix 1 [file mhealth_v13i1e68665_app1.docx]

| Supplemental Table S1. Differences between the Headspace Sleep Program, sleep mindfulness content, and cognitive behavioral therapy for insomnia. | | | | |
| --- | --- | --- | --- | --- |
|  | Digital mindfulness sleep content | Headspace Sleep Program | Digital CBT-I | In-person CBT-I |
| Overview | Digital content dedicated to finding restful sleep | Sequential digital content dedicated to improving sleep quality (the current intervention) | Cognitive behavioral therapy (CBT) for insomnia to improve sleep quality, accessed via digital app | Cognitive behavioral therapy (CBT) for insomnia to improve sleep quality, accessed in-person |
| What is it? | Mindfulness techniques and breathwork designed to prepare the mind for sleep, stories and sounds to help you get and stay asleep | Mindfulness techniques and CBT-I principles (no sleep restriction) | Behavioral techniques, cognitive strategies, and de-arousal strategies to improve sleep (includes sleep restriction) | Behavioral techniques, cognitive strategies, and de-arousal strategies to improve sleep (includes sleep restriction) |
| How often? | One off content to be accessed before/while going to sleep | 18 consecutive, daily sessions | 6-8 weeks of weekly, digital sessions | 6-8 weeks of weekly, in-person sessions |
| Who leads? | Self-guided, content lead by Headspace mindfulness teachers | Self-guided, sessions lead by a mindfulness teacher, sleep expert, and Headspace members | Clinician | Clinician |
| Where is it located? | Headspace app | Headspace app | Digital, scheduled sessions | In-person, scheduled sessions |
| What is the cost? | Headspace membership  *$12.99/month or $69.99/year* | Headspace membership  *$12.99/month or $69.99/year* | *$99-$600 [58]* | Per session cost  *Up to $250/session depending on insurance [59, 60]* |
| Who is the target? | Anyone | Anyone, but designed to deliver outcomes for subclinical/clinical sleep disturbance | Clinical levels of sleep disturbance | Clinical levels of sleep disturbance |
| Abbreviations: CBT-I (cognitive behavioral therapy for insomnia) | | | | |
