# Supplementary material for: Assessment of an App-Based Sleep Program to Improve Sleep Outcomes in a Clinical Insomnia Population: Randomized Controlled Trial
Source: JMIR Mhealth Uhealth. 2025 Apr 23;13:e68665. doi: 10.2196/68665 (PMC12059489; doi:10.2196/68665)
Supplement: Multimedia Appendix 2 [file mhealth_v13i1e68665_app2.docx]

| Supplemental Table S2. Headspace Sleep Program session outline and descriptions. | |
| --- | --- |
| **Session** | **Session objective** |
| Part 1 | Introduces mindfulness as a way to observe sleep experience and develop insights into what factors may be driving it |
| Session 1 | Introduces the program and exploration of deeper motivations for improving sleep |
| Session 2 | Introduces mindfulness as a way to non-judgmentally observe sleep experience |
| Session 3 | Describes how thoughts can impact sleep, and introduces mindfulness as a tool for observing thoughts |
| Session 4 | Describes how behaviors can impact sleep, and introduces mindfulness as a tool for observing thoughts |
| Part 2 | Builds upon mindfulness tools to support learning and experimentation with evidence-based tools for improving sleep (i.e., adding CBT-I techniques to mindfulness tools) |
| Session 5 | Introduces stimulus control tool (i.e., building a connection between being in bed and being asleep by staying out of bed if awake and not sleepy) |
| Session 6 | Introduces sleep window tool (i.e., waking up at the same time every day and only going to bed when sleepy, or a bit later than usual) |
| Session 7 | Introduces sleep hygiene tools (i.e., behaviors that regulate the sleep and wake drives, such as reducing stimulant intake before bedtime, getting bright light exposure in the morning, etc) |
| Session 8 | Introduces bedtime ritual as a tool (i.e., routines that help them wind down before bed and further associate the bed with sleepiness) |
| Session 9 | Introduces a progressive muscle relaxation tool (i.e., tensing muscles before relaxing them to help reduce tension and get better at entering a relaxed state) |
| Session 10 | Introduces a tool for thinking flexibly (i.e., recognizing that not all thoughts are helpful and practicing taking new perspectives) |
| Session 11 | Introduces a tool for managing worry (i.e., practicing worrying in a specific time and place and delaying worry outside of that time and place) |
| Part 3 | Helps members learn behavior change strategies to take tools they’ve learned and continue practicing them (i.e., implementing mindfulness and CBT-I tools into daily practice) |
| Session 12 | Describes basic habit formation skills |
| Session 13 | Describes problem solving skills |
| Session 14 | Describes strategies for maintaining motivation |
| Session 15 | Describes effective communication strategies for building support |
| Session 16 | Describes strategies for handling setbacks |
| Session 17 | Describes strategies for planning for the future |
| Session 18 | Concludes program, describe sleep as an ongoing journey that requires continued practice |
