# Supplementary material for: Assessment of an App-Based Sleep Program to Improve Sleep Outcomes in a Clinical Insomnia Population: Randomized Controlled Trial
Source: JMIR Mhealth Uhealth. 2025 Apr 23;13:e68665. doi: 10.2196/68665 (PMC12059489; doi:10.2196/68665)
Supplement: Multimedia Appendix 3 [file mhealth_v13i1e68665_app3.docx]

**Supplementary Results - Study-Completer analysis**

In the main analysis we report results from an intention-to-treat (ITT), however we also report results on study-completer analyses (SC). The ITT population included all randomly allocated participants, whereas the SC population included all participants who did not drop out of the study (see Figure 1). Below, we report SC analyses.

**Results**

The 117 participants included in the study-completer analysis had a mean (SD) age of 37.3 (11.1) years and 54.7% (64 females) identified as female (Table S3). No significant differences were identified between the intervention and control groups at baseline for demographic characteristics or study outcomes. On average, participants in the intervention group completed 14.1 sessions (SD=4.8), corresponding to 78.3% of the intervention content. A total of 58/58 (100%) participants completed at least 2 sessions of the app-based Sleep Program. A total of 24/58 (50.0%) participants completed all 18 sessions of the intervention and 51/58 (87.9%) completed at least half of the intervention. Medication status at baseline for the intervention group showed that a total of 15/58 (25.8%) participants took sleep medication on a regular basis, and 43/58 (74.2%) participants did not use sleep medication. For the control group 16/59 (27.1%) participants took sleep meditation on a regular basis at baseline, whereas 43/59 (72.9%) did not. At follow-up there was no change in either group in usage of sleep medication. There were no significant differences between the two groups at baseline or follow-up, and no changes within either group (Table S3).

**Insomnia Outcomes**

ISI results indicated a group x time interaction (P<.001, η²p=0.24) with the sleep program group significantly decreasing ISI from pre- to post-intervention (20.52% decrease, P<.001), pre-intervention to follow up (28.94% decrease, P<.001), and post-intervention to follow up (10.60% decrease, P=.01). No significant changes were identified in the control group (P>.05) (Table S4).

Remission rates (ISI total score <8) [26,27] in the intervention group increased significantly over time (11.9% at post-intervention (6 participants over 58) and 13.5% at follow-up (7 participants over 58) while it was unchanged in the control group (1.5% at post-intervention (1 participants over 59) and 0% at follow-up). Similarly, treatment response (ISI total score reduction >7) [26,27] showed a steady increase from pre- to post-intervention (18.5%, 10 participants over 58) to follow-up (23.8%, 13 participants over 58) in the intervention group while it did not significantly change in the control group (4.9% at post-intervention (3 participants over 59) and 1.5% at follow-up (1 participant over 59)).

Sleep diary results suggest that the Headspace Sleep Program group significantly increased SE from pre- to post-intervention (P=.001) and pre-intervention to follow-up (P=.01), but not from post-intervention to follow-up (P=.76) (Table S5). A group x time interaction was identified (P=.01, η²p=.04). No significant changes in SE were identified in the control group (P>.05). There were no group x time interactions for SOL (P=.26, η²p=.01), WASO (P=.14, η²p=.02), and TST (P=.07, η²p=.02). For TST, a significant main effect of group (P=.04, η²p=.04) showed that the groups were significantly different without considering the time points from pre- to post-intervention to follow-up (Table 3). No main effects of time were identified for SOL (P=.15, η²p=.01) and WASO (P=.23, η²p=.03). A significant main effect of group for SOL (P=.02, η²p=.04) showed that both groups increased over time from pre- to post-intervention to follow-up (Table S5).

Objective sleep quality actigraphy results yielded similar results to the subjective sleep diary results. A group x time interaction was identified for SE (P<.001, η²p=.10), SOL (P<.001, η²p=.07), and WASO (P=.01, η²p=.04), but not for TST (P=.07, η²p=.02). The Headspace Sleep Program group significantly improved SE, SOL, and WASO with results showing no significant improvements in the control group (Table S6). For TST, a significant main effect of group (P=.02, η²p=.04) showed that the groups were significantly different without considering the time points. (Table S6).

Subgroup analysis compared mild versus moderate baseline insomnia symptoms (mild: ISI=8-14, 50 subjects; moderate: ISI=15-21, 67 subjects) with ANOVA results showing a significant three-way interaction (P=.03, ηp2 =.03) and follow-up testing revealing that those with moderate baseline insomnia symptoms in the Headspace Sleep Program group improved ISI significantly from pre- to post-intervention (P<.001), pre-intervention to follow-up (P<.001), and post-intervention to follow-up (P=.04). Those with mild baseline insomnia symptoms in the Headspace Sleep Program group did not show a significant change from pre-intervention to post-intervention (P=.85); But did show a significant improvement from pre-intervention to follow-up (P=.045) and from post-intervention to follow-up (P=.04).

**Mental Health Outcomes**

Group x time interactions were identified for PHQ-8 (P=.003, η²p=.05), PSQI (P<.001, η²p=11), GAD-7 (P<.001, η²p=.15), and MAAS (P=.02, η²p=.07). The PHQ-8, PSQI, GAD-7, and MAAS results showed that the Headspace Sleep Program group significantly improved at all time points (P<.05) and the control group did not significantly change at any time point (P>.05). PSS-10 results did not show any group x time interaction (P=.79, η²p=.01) or main effect of group (P=.21, η²p=.01). A significant main effect of time (P<.001, η²p=.06) showed that both groups increased PSS-10 over time from pre- to post-intervention to follow-up (Table S4).

Subgroup analysis ANOVA results showed no significant three-way or two-way interactions for PSS-10. A main effect of time is shown (P=.002). Similarly, no significant three-way or two-way interactions were shown for PHQ-8. A main effect of time (P<.001) and group were present (P=.002). No significant three-way or two-way interactions were shown for PSQI. A main effect of time (P= .01) was present. No significant three-way or two-way interactions were shown for GAD-7. A main effect of time (P<.001) and group were present (P=.008). ANOVA results showed a significant three-way interaction for MAAS (P=.03 ηp2 =.034) with follow-up testing revealing that the moderate insomnia group in the experimental condition improved MAAS significantly from pre- to post-intervention (P=.002) and follow-up (P<.001) but not from post-intervention to follow-up (P=.132). By contrast, the mild insomnia group in the experimental group did not show a significant change from pre-intervention to post-intervention (P=.587) or follow-up (P=.061). However, a significant difference from post-intervention to follow-up (P=.023) was observed. No significant differences were reported for both moderate and mild insomnia in the waitlist control at any time point.

| Table S3. Demographics and clinical characteristics by study group of study-completer sample. | | |
| --- | --- | --- |
|  | Sleep Intervention (N=58) | Waitlist Control (N=59) |
| Gender, n (%) |  |  |
| Male | 28 (48.3) | 25 (42.4) |
| Female | 30 (51.7) | 34 (57.4) |
| Age, mean (SD) | 37.6 (12.2) | 37.1 (10.1) |
| Marital Status, n (%) |  |  |
| Married | 22 (37.9) | 22 (37.3) |
| In a relationship | 10 (17.2) | 11 (18.6) |
| Single | 18 (31.1) | 20 (33.9) |
| Divorced | 8 (13.8) | 6 (10.2) |
| Employment, n (%) |  |  |
| Full time | 31 (53.4) | 30 (50.9) |
| Part time | 15 (25.9) | 11 (18.6) |
| Retired | 2 (3.5) | 4 (6.8) |
| Unemployed | 10 (17.2) | 14 (23.7) |
| Household Income, n (%), $USD |  |  |
| <20,000 | 6 (10.3) | 9 (15.2) |
| 20-35,000 | 12 (20.7) | 10 (17) |
| 35-50,000 | 12 (20.7) | 13 (22) |
| 50-75,000 | 10 (17.3) | 8 (13.6) |
| 75 -100,000 | 8 (13.7) | 10 (17) |
| >100,000 | 10 (17.3) | 9 (15.2) |
| Ethnicity, n (%) |  |  |
| White | 31 (53.5) | 31 (54.2) |
| Asian | 6 (10.3) | 7 (11.9) |
| Black | 9 (15.5) | 10 (17) |
| Hispanic | 7 (12.1) | 6 (10.1) |
| Other | 5 (8.6) | 4 (6.8) |
| Sleep Medication, n (%) |  |  |
| Yes (Baseline)  No (Baseline) | 15 (25.8)  43 (74.2) | 16 (27.1)  43 (72.9) |
| Yes (Follow-up)  No (Follow-up) | 15 (25.8)  43 (74.2) | 16 (27.1) 43 (72.9) |

| Table S4. Behavioral data by study group and timepoint of study-completer sample. | | | | | | |
| --- | --- | --- | --- | --- | --- | --- |
|  | Sleep Intervention (N=58) | | | Waitlist Control (N=59) | | |
| Clinical Value | Pre | Post | Follow-up | Pre | Post | Follow-up |
| ISI, mean (SD) | 14.96 (3.10) | 11.89 (4.05)  †c | 10.63 (3.13)  ‡c  §b | 15.08 (2.96) | 15.25 (4.47) | 15.05 (3.21) |
| PSQI, mean (SD) | 10.51 (2.43) | 9.46 (2.20)  †c | 8.08 (2.42)  ‡c  §b | 10.59 (2.67) | 10.71 (2.25) | 10.20 (1.81) |
| PSS-10, mean (SD) | 17.44 (3.36) | 18.74 (3.27) | 18.51 (3.24) | 17.03 (3.80) | 17.90 (2.98) | 17.93 (2.47) |
| PHQ-8, mean (SD) | 11.46 (3.95) | 10.03 (4.52)  †c | 8.98 (4.13) ‡c  §b | 11.75 (3.51) | 11.69 (2.63) | 11.07 (2.75) |
| GAD-7, mean (SD) | 11.01 (4.61) | 8.68 (4.14)  †c | 8.03 (4.38) ‡c  §a | 10.90 (4.10) | 11.41 (3.55) | 11.34 (3.38) |
| MAAS, mean (SD) | 50.39 (16.64) | 53.24 (15.74)  †b | 55.06 (14.76)  ‡c  §b | 50.69 (14.15) | 50.14 (12.31) | 50.76 (11.37) |
| Abbreviations: ISI (insomnia severity index); PSS-10 (perceived stress scale); PHQ-8 (Patient Health Questionnaire); GAD-7 (Generalized Anxiety Disorder); MAAS (Mindful Attention Awareness Scale).  ap<0.05  bp<0.01  cp<.001  †: Significant difference between pre and post timepoints  ‡: Significant difference between pre and follow-up timepoints  §: Significant difference between post and follow-up timepoints | | | | | | |

| Table S5. Subjective measures of sleep (i.e., sleep diary) by study group and timepoint of study-completer sample. | | | | | | |
| --- | --- | --- | --- | --- | --- | --- |
|  | Sleep Intervention (N=58) | | | Waitlist Control (N=59) | | |
| Clinical Value | Pre | Post | Follow-up | Pre | Post | Follow-up |
| SE, mean (SD), % | 75.27 (17.57) | 83.29 (9.37)  †a | 82.62 (13.24) ‡b | 77.89 (13.23) | 76.14 (13.90) | 78.28 (9.40) |
| SOL, mean (SD), minutes | 36.96 (22.01) | 27.15 (21.51) | 25.72 (29.87) | 38.05 (29.79) | 39.37 (38.83) | 35.41 (25.54) |
| WASO (SD), minutes | 88.81 (88.62) | 57.10 (52.93) | 66.93 (78.25) | 75.49 (73.15) | 79.69 (65.96) | 68.71 (62.40) |
| TST, mean (SD), minutes | 384.91 (111.54) | 404.96 (78.44) | 410.03 (104.09) | 392.98 (107.59) | 380.58 (95.47) | 359.73 (78.94) |
| Abbreviations: SE (sleep efficiency); SOL (sleep onset latency); WASO (wake after sleep onset); TST (total sleep time).  ap<0.05  bp<0.01  †: Significant difference between pre and post timepoints  ‡: Significant difference between pre and follow-up timepoints  §: Significant difference between post and follow-up timepoints | | | | | | |

| Table S6. Objective measures of sleep (i.e. actigraphy) by study group and timepoint of study-completer sample. | | | | | | |  |
| --- | --- | --- | --- | --- | --- | --- | --- |
|  | Sleep Intervention (N=58) | | | Waitlist Control (N=59) | | |  |
| Clinical Value | Pre | Post | Follow-up | Pre | Post | Follow-up |  |
| SE, mean (SD), % | 74.72 (7.38) | 82.70 (8.12)  †c | 82.96 (9.42) ‡c | 77.94 (9.01) | 75.62 (12.61) | 75.64 (10.83) |  |
| SOL, mean (SD), minutes | 49.20 (23.49) | 29.18 (19.06) †c | 26.38 (21.13) ‡c | 39.32 (24.50) | 40.93 (37.61) | 42.98 (24.71) |  |
| WASO (SD), minutes | 79.78 (48.29) | 56.50 (47.40) †a | 58.08 (52.48) ‡a | 68.78 (51.26) | 79.63 (63.84) | 71.03 (46.33) |  |
| TST, mean (SD), minutes | 377.00 (61.73) | 394.52 (51.32) | 392.55 (56.68) | 383.83 (73.48) | 376.00 (88.90) | 356.39 (68.94) |  |
| Abbreviations: SE (sleep efficiency); SOL (sleep onset latency); WASO (wake after sleep onset); TST (total sleep time).  ap<0.05  bp<0.01  cp<.001  †: Significant difference between pre and post timepoints  ‡: Significant difference between pre and follow-up timepoints  §: Significant difference between post and follow-up timepoints | | | | | | |  |
