# Supplementary figures and images for: Assessment of an App-Based Sleep Program to Improve Sleep Outcomes in a Clinical Insomnia Population: Randomized Controlled Trial
Source: JMIR Mhealth Uhealth. 2025 Apr 23;13:e68665. doi: 10.2196/68665 (PMC12059489; doi:10.2196/68665)

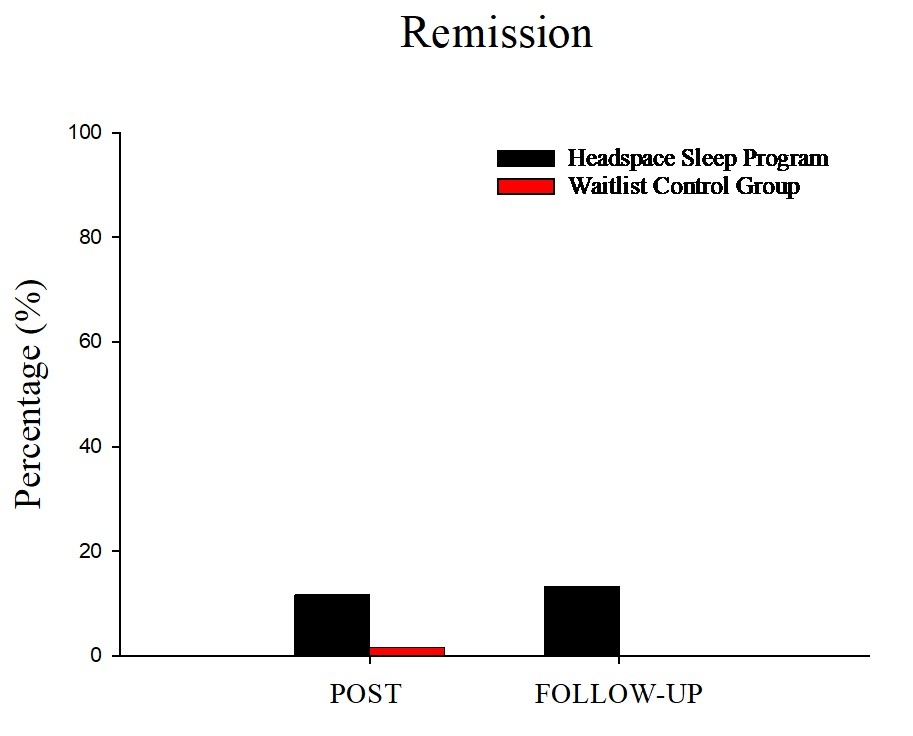

Supplement: Multimedia Appendix 5 [file mhealth_v13i1e68665_app5.png]

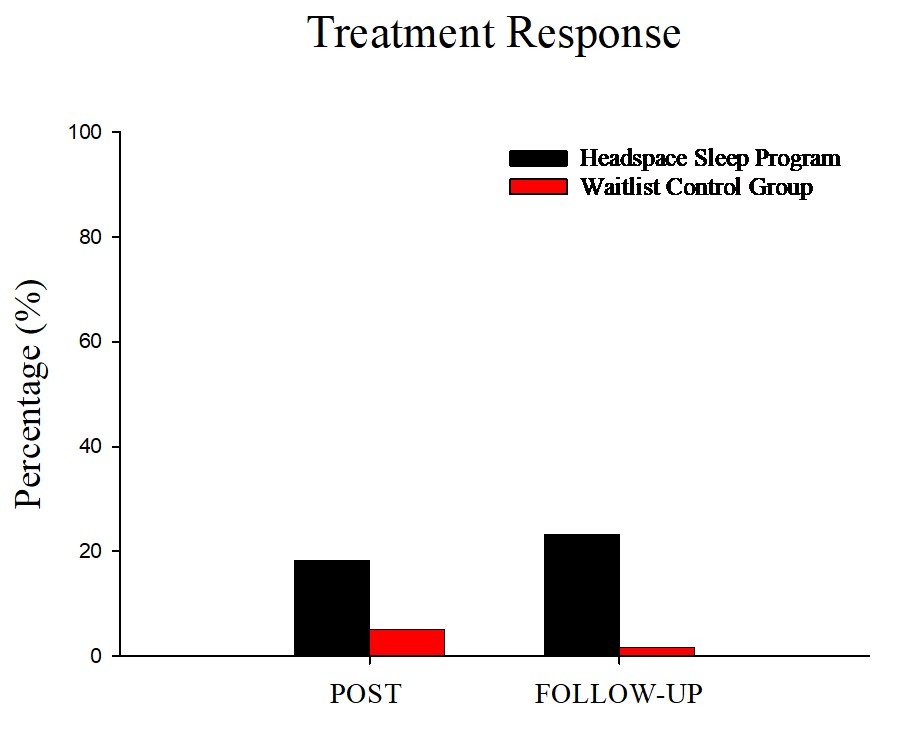

Supplement: Multimedia Appendix 6 [file mhealth_v13i1e68665_app6.png]

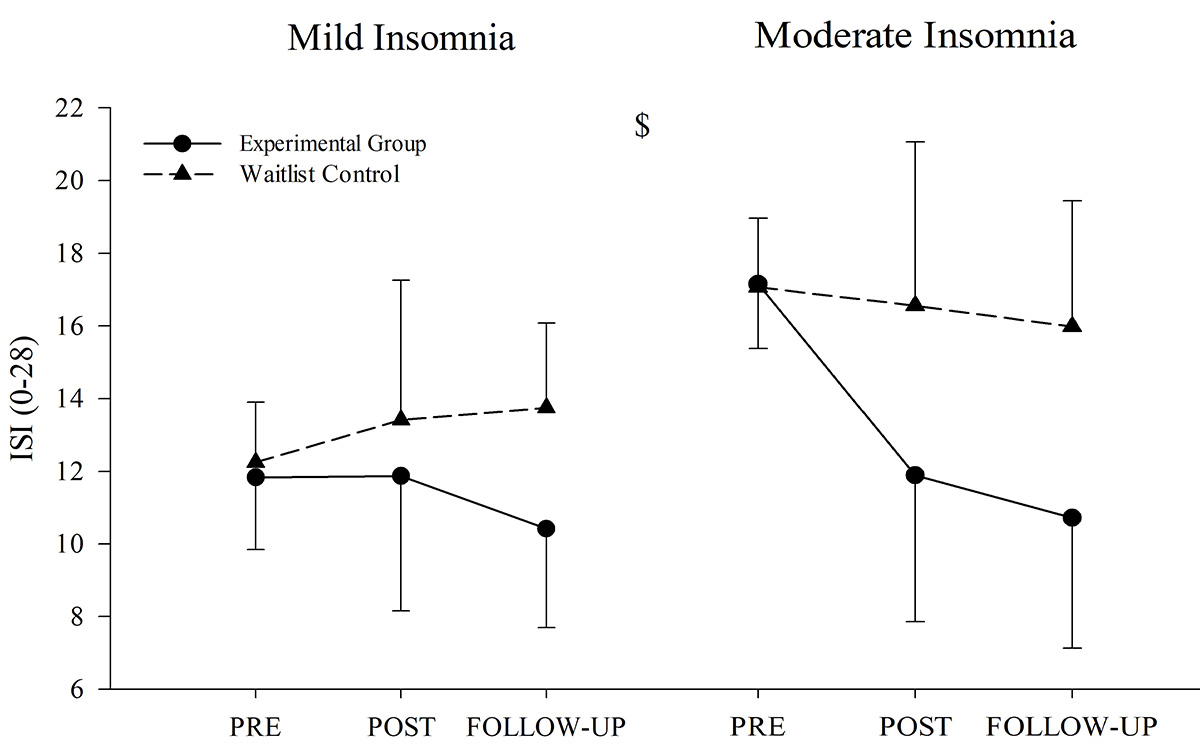

Supplement: Multimedia Appendix 7 [file mhealth_v13i1e68665_app7.png]
